# Comparing benign to malignant cystectomy: complications, emergency department utilization, readmissions, and socioeconomic status

**DOI:** 10.1007/s11255-025-04972-7

**Published:** 2025-12-25

**Authors:** Maxwell Sandberg, David Thole, Randall Bissette, Dylan Wolff, Madeline Sandberg, Lindsay Schwartz, Ethan Weitzman, Robert Evans, Alejandro Rodriguez

**Affiliations:** 1 Wake Forest University School of Medicine, Winston Salem, NC USA; 2 University of Michigan, Ann Arbor, MI USA; 3 Wake Forest Comprehensive Cancer Center, Winston Salem, NC USA

**Keywords:** Benign, Cystectomy, Surgery, Emergency department, Readmission

## Abstract

**Introduction:**

Research has often focused on radical cystectomy for bladder cancer; however, there is an entirely different population, namely those who undergo cystectomy for benign, non-cancerous reasons, which requires attention. The purpose of this study was to compare patients who underwent cystectomy for benign (BC) and malignant (MC) reasons, focusing on complications, emergency department (ED) utilization, readmission, and socioeconomic status (SES).

**Methods:**

Cystectomies performed at our institution from 2012 to 2025 were reviewed. Patients were divided based on indication into BC versus MC (for bladder cancer with pathologic confirmation). Non-oncologic cystectomies were either for neurogenic bladder, interstitial cystitis/bladder pain syndrome, or “other reasons.” Complications were divided into in-house immediately following cystectomy prior to discharge and those occurring within 90 days of discharge. The Memorial Sloan Kettering Cancer Center classification system of cystectomy complications was used to qualify complications. ED utilization and readmissions were charted within the first 90 days of discharge. Area Deprivation Index (ADI) scores, which assign rankings to SES for neighborhoods, were used as a surrogate for patient SES. BC and MC patients were compared.

**Results:**

569 patients were included in the analysis (183 BC and 386 MC). Urinary diversion choice differed between groups, with more cutaneous ureterostomies in MC patients and Indiana Pouches in BC patients (*p* < 0.001). In-house complications were significantly greater in the benign cohort (51%) compared with the malignant cohort (36%; *p* < 0.001). ED utilization within 90 days of discharge was greater in BC (51%) relative to MC (34%; *p* < 0.001) patients, and BC patients were more likely to visit the ED with no readmission (*p* < 0.001). 69 (40%) patients were readmitted in the BC group, and 67 (17%; *p* < 0.001) patients were readmitted in the MC group. ADI scores 1–4 (higher SES) were more common in BC patients (*p* = 0.012).

**Conclusions:**

BC patients have greater rates of healthcare utilization compared to those undergoing MC. While recognizing these patient populations are not the same, the causes of these differences call for additional investigation.

**Supplementary Information:**

The online version contains supplementary material available at 10.1007/s11255-025-04972-7.

## Introduction

Cystectomy involves the surgical removal of all or part of the bladder and is traditionally performed by urologists in patients with oncologic disease, most commonly bladder cancer. Prior research has often focused on either bladder-sparing treatments with trimodal therapy (transurethral resection of bladder tumor, chemotherapy, and radiation) or radical cystectomy (RC) for bladder cancer to identify optimal surgical techniques, perioperative morbidity and mortality, along with postoperative outcomes [1]. Evidence indicates that RC for bladder cancer carries a complication rate of approximately 60% within 90 days of surgery [2, 3]. Further, emergency department (ED) utilization and readmission rates within 90 days of RC are both high, ranging from 7 to 34% and 36–39%, respectively [4–6]. The significant morbidity and its clinical impact have been extensively researched. This is certainly important given that some studies estimate approximately 95% of cystectomies are performed for bladder cancer [7, 8]. However, there is an entirely different patient population, namely those who undergo cystectomy for benign, non-cancerous reasons, which requires additional attention.

Benign cystectomy (BC) is estimated to make up 5.4–8% of all cystectomies performed in the United States [7–9]. This is, by definition, surgical removal of the bladder for reasons other than oncologic disease. This includes indications like urogenital fistula, radiation cystitis, interstitial cystitis/bladder pain syndrome (IC/BPS), stricture disease, refractory urinary incontinence, and neurogenic bladder, but is not limited to this [8]. Further, guidelines for IC/BPS and neurogenic bladder both list BC as an evidence-based surgical procedure [8, 10, 11]. There are a multitude of surgical approaches and techniques to perform BC. Most often, a sub-trigonal cystectomy (SubTC) is performed, where the bladder, trigone, and ureteral orifices are removed, or a supra-trigonal cystectomy (SupTC) is performed, where the bladder is divided above the trigone, leaving ureteral orifices in place [12]. Like in RC for malignant disease, urinary diversion varies based on both surgeon and patient preference.

Complication rates and the types of complications patients may have postoperatively from BC are not as well understood. Some approximate that 35–39% of patients undergoing BC will experience a postoperative complication [13, 14]. Even fewer publications compare BC to RC for malignant disease. One such study did not identify differences in morbidity nor mortality between the two groups [9]. Additionally, questions remain about the patient populations undergoing BC versus RC for bladder cancer in terms of their socioeconomic status (SES). Yet, the literature comparing BC to RC for bladder cancer leaves much to be desired. The purpose of this study was to compare BC to RC for malignancy, focusing on postoperative complications, ED utilization, readmission rates, and SES.

## Methods

This was a retrospective analysis of all cystectomies performed at our institution from 2012 to 2025. Patients were divided into those who underwent cystectomy for oncologic (malignant) versus benign indications. All oncologic cystectomies had a pathologically confirmed diagnosis of bladder cancer. Non-oncologic cystectomies (BC) were either for IC/BPS, neurogenic bladder, or “other reasons.” Other reasons included radiation cystitis, refractory urinary incontinence, and bladder fistula. A variety of patient demographic data were collected like age at surgery, self-identified race and gender, comorbidities, and Charlson Comorbidity Index (CCI). Cystectomy approach was either open or robotic, and then surgical format was further divided into RC, SubTC, or SupTC. Oncologic patients that did not undergo RC for their bladder cancer were excluded, as were BC patients whose final pathology showed any form of cancer in the bladder. Additional exclusion criteria included benign patients who underwent urinary diversion but not BC, and patients missing pathologic data postoperatively. There were a minority of BC patients who underwent RC, and these patients were still included in the final analysis if their final pathology revealed no evidence of bladder cancer.

Complications were divided into those that occurred in-house immediately following cystectomy prior to discharge and those occurring within 90 days of discharge from cystectomy that required presentation to an urgent care, ED, or readmission. The Clavien–Dindo (Clavien) classification system was used to classify complications, and the Memorial Sloan Kettering Cancer Center (MSKCC) classification system of cystectomy complications was used to qualify the type of complications patients had after their surgery [15, 16]. The MSKCC classification system is a validated way to qualify the types of postoperative complications patients experience after cystectomy. This included infectious, bleeding, gastrointestinal (GI), genitourinary (GU), wound, cardiac, pulmonary, thromboembolic, neurological, surgical, and miscellaneous/other complications. Patients could have had multiple MSKCC complications at the same time. Similarly, ED utilization was charted within the first 90 days of discharge from cystectomy. Readmissions within 90 days of discharge from cystectomy were recorded as was the reason for readmission using the MSKCC system. Survival and most recent follow-up dates were noted. Each patient also had their area deprivation index (ADI) score calculated to serve as a surrogate for SES [17, 18]. ADI is an independently validated scoring system to rank neighborhood SES disadvantage in the United States of America. The score combines multiple factors including income, education, employment, and housing conditions to develop a neighborhood SES [19]. Worse ADI scores mean lower SES and have been linked to poor health outcomes in a multitude of previous publications [20]. DD was either home, home with nursing assistance, or skilled nursing facility (SNF), and a comprehensive evaluation by physical therapy made the final recommendations on this. All chart reviewed data were independently reviewed and verified by at least two authors on this manuscript.

Chi-squared tests and independent samples t test were performed to compare patients based on whether their cystectomy was for benign or malignant reasons. A multinomial logistic regression model was used to compare Clavien classification complications between benign and malignant patients where applicable, MSKCC complications, and ADI scores. Further, a binary logistic regression model was developed with BC or RC for malignancy as the outcome. The model was run as a backward regression at a significance of *p* < 0.05 and produced an optimized model fit without collinearity between variables. Variables in the model were based on univariable analysis *p* < 0.1 and/or clinical relevance, and adjusted odds ratios (OR) were calculated. Additionally, patients undergoing RC for malignant reasons were subdivided based on pathologic stage ≤ pT2 versus > pT2 and were compared to BC patients to assess the rate of immediate in-house complications, ED utilization, and readmission to assess the role that pathologic stage may have played.

## Results

There was a total of 569 cystectomy patients included in the analysis (183 BC and 386 malignant; Table 1). Mean age at surgery was significantly lower in the benign group (57 years) than the malignant grouping (67 years; *p* < 0.001). Women made up 69% of the benign cohort and 20% of the malignant cohort (*p* < 0.001). Self-identified race was not significantly different between the two groups (*p* > 0.05). Prevalence of hypertension, diabetes mellitus, and coronary artery disease were alike in both groups (*p* > 0.05). Chronic Obstructive Pulmonary Disease was more prevalent in the malignant cohort (13%) than the benign cohort (4%; *p* = 0.002). Mean CCI was greater in the malignant cohort (5) compared to the benign cohort (3; *p* < 0.001). In those who underwent BC, 168 (92%) had a SubTC, 12 (6%) had a SupTC, and 3 (2%) had a RC. Operative time was not significantly different between benign (347 min) and malignant (349 min; *p* > 0.05) patients. Likewise, length of stay was not different between benign (7 days) and malignant (14 days; *p* > 0.05) patients. All BCs were performed with an open approach, and 219 (57%) RCs were performed open, with 167 (43%) done robotic. Urinary diversion choice differed between the groups (*p* < 0.001). There were 140 (77%) ileal conduits, 16 (9%) Indiana pouches, 26 (14%) neobladders, 0 cutaneous ureterostomies (CUs), and 1 (0.5%) percutaneous nephrostomy tube (PCN) in the benign cohort. There were 317 (82%) ileal conduits, 1 (0.3%) Indiana pouch, 8 (2%) neobladders, 50 (13%) CUs, and 10 (3%) PCNs in the malignant cohort. A Bricker anastomosis was used 11% of the time in ileal conduits for benign patients compared to 70% (*p* < 0.001) in ileal conduits for malignant patients. A total of 237 (61%) malignant patients had pathological stage ≤ pT2 that underwent RC.

**Table 1:** Comparison of benign and malignant patients

Variable	Benign (*n* = 183)	Malignant (*n* = 386)	*P* value
Age (years)	57 ± 16	67 ± 10	< 0.001
Female	127 (69)	78 (20)	< 0.001
Race	–	–	0.33
White	160 (87)	337 (87)	–
Black	13 (7)	38 (10)	–
Hispanic	4 (2)	3 (1)	–
Asian	2 (1)	3 (1)	–
Native American	1 (1)	4 (1)	–
Pacific Islander	1 (1)	0 (0)	–
Other	2 (1)	1 (1)	–
CCI	3 (2)	5 (2)	< 0.001
Diabetes	45 (25)	92 (24)	0.844
Hypertension	87 (48)	203 (53)	0.26
Coronary artery disease	35 (19)	70 (18)	0.776
Chronic obstructive pulmonary disease	8 (4)	49 (13)	0.002
Subtrigonal	168 (91)	0	< 0.001
Supratrigonal	12 (7)	0	
Radical	3 (2)	386 (100)	
Urinary diversion	–	–	< 0.001
Ileal conduit	140 (77)	317 (82)	–
Indiana pouch	16 (9)	1 (1)	–
Neobladder	26 (14)	8 (2)	–
Cutaneous U	0 (0)	50 (13)	–
PCN	1 (1)	9 (2)	–
Open surgery	183 (100)	219 (57)	< 0.001
Anastomosis type	–	–	< 0.001
Bricker	15 (4)	272 (86)	–
Wallace	125 (96)	45 (14)	–
ADI	–	–	0.012
1	10 (5)	10 (3)	–
2	17 (9)	23 (6)	–
3	18 (10)	18 (5)	–
4	26 (14)	41 (11)	–
5	24 (13)	49 (13)	–
6	21 (12)	41 (11)	–
7	19 (10)	58 (15)	–
8	17 (9)	54 (14)	–
9	18 (10)	38 (10)	–
10	12 (7)	52 (14)	–
Discharge destination			0.360
Home	90 (49)	211/382 (55)	
HNA	78 (43)	147/382 (38)	
SNF	15 (8)	24/382 (7)	
Operative time (min)	347.4 ± 87.1	349 ± 93.5	0.84
LOS (Days)	7.2 ± 4.8	13.8 ± 113.2	0.428
Died before ever being discharged	2 (1)	4 (1)	0.951
Immediate postoperative complication	92 (50)	139 (36)	< 0.001
Immediate postoperative complication, Clavien grade	–	–	< 0.001
I	15 (16)	59 (43)	–
II	47 (51)	45 (33)	–
III	14 (15)	8 (6)	–
IV	2 (2)	6 (4)	–
V	10 (11)	3 (2)	–
VI	4 (4)	12 (9)	–
VII	1 (1)	4 (3)	–
ED visit within 90 days of discharge	92 (50)	130 (34)	< 0.001
If yes, how many ED visits?	1.7 ± 1.1	1.5 ± 1.0	0.213
ED visit, no readmission	36 (41)	29 (8)	< 0.001
Readmitted within 90 days	69 (40)	67 (17)	< 0.001
Readmission, Clavien grade	–	–	0.261
I	10 (11)	10 (15)	–
II	42 (47)	42 (63)	–
IIIa	15 (17)	6 (9)	–
IIIb	13 (14)	5 (7)	–
IVa	5 (6)	2 (3)	–
IVb	3 (3)	2 (3)	–
V	2 (2)	0 (0)	–
Post-op antibiotics given	60 (33)	6 (86)	0.004
UTI within 90-days	35 (19)	25 (7)	< 0.001
Stricture developed	34/181 (19)	7 (26)	0.384
Dead	25 (14)	171 (44)	< 0.001
Follow-up (months)	39.1 ± (37.2)	26.7 ± 81.2	0.051

The following table compares benign cystectomy and malignant cystectomy patients in the study population. Variables are listed in the left-most column. Continuous variables are means with standard deviations and categorical variables are totals with percentage of the cohort in parentheses. *P* values are provided for each variable comparison in the left-most column

On binary logistic regression (Supplementary Table 1), a greater CCI score (OR 1.7 per 1 unit increase), female gender (OR 7), postoperative complications (OR 2.8), readmission within 90 days of discharge (OR 4.1), and a continent diversion (OR 5.1) were more common in BC patients (*p* < 0.05; Supplementary Table 1). ADI scores 1–4 were significantly less likely in BC patients (*p* < 0.05).

The rate of in-house complications was significantly greater in the benign cohort (51%) compared with the malignant cohort (36%; *p* < 0.001). The three most common MSKCC complication types were GI (*N* = 39; 37%; Table 2), GU (*N* = 26; 25%), and infectious (*N* = 12; 11%) in the BC cohort. In the malignant RC grouping, they were GI (*N* = 57; 32%), GU (*N* = 35; 20%), and cardiac (*N* = 23; 13%). The types of complications patients experienced in-house were significantly different (*p* = 0.01). Specifically, malignant patients were 26.3 times more likely to have a GU complication postoperatively on multinomial logistic regression and 5.8 times more likely to have a cardiac complication (Supplementary Table 2). ED utilization within 90 days of discharge was greater in benign patients (51%) relative to malignant (34%; *p* < 0.001) patients, but the mean number of ED visits did not differ (*p* > 0.05). BC patients were more likely to visit the ED and not be readmitted (39%) compared to malignant patients (22%; *p* < 0.001). When dividing malignant patients based on pathological stage, BC patients (51%) still carried a greater rate of in-house complications regardless of whether pathological stage was ≤ pT2 (37%; *p* = 0.005) or > pT2 (34%; *p* = 0.002). ED visits were also more likely in BC patients (51%) regardless of patient pathological stage ≤ pT2 (32%; *p* < 0.001) or stage > pT2 (37%; *p* = 0.010).

**Table 2:** Immediate postoperative complication classifications

Category	UTI/infection	Bleeding	Surgical	GU	GI	Wound	DVT/PE	Cardiac	Other	Neurologic	Pulmonary	Total
Benign	12 (11.3)	26 (24.5)	2 (1.9)	2 (1.9)	39 (36.8)	8 (7.5)	0	6 (5.7)	6 (5.7)	4 (3.8)	1 (0.9)	106
Malignant	14 (7.9)	10 (5.6)	6 (3.4)	35 (19.7)	57 (32.0)	12 (6.7)	4 (2.2)	23 (12.9)	4 (2.2)	7 (3.9)	6 (3.4)	178
Total	26	36	8	37	96	20	4	29	10	11	7	284

The following table categorizes immediate postoperative complications after cystectomy between benign and malignant patients using the MSKCC Classification System. Total numbers with percentage of the total cohort in parentheses are displayed. As there were no DVT/PE complications in the benign cohort, these complications were excluded from the model

A total of 69 (40%) patients were readmitted in the benign group and 67 (17%; *p* < 0.001) patients were readmitted in the malignant group within 90 days of discharge. Clavien IVa complications were 92% more likely in BC patients on multinomial regression (Supplementary Table 3). The three most common complications associated with readmission in the BC group were UTI/infectious (*N* = 28; 37%; Table 3), GU (*N* = 13; 17%), and wound-related (*N* = 12; 16%). The three most common complications associated with readmission in the malignant group were UTI/infectious (*N* = 29; 37%; Table 3), GU (*N* = 16; 20%), and GI (*N* = 13; 16%). Malignant patients were 28 times more likely to experience a pulmonary complication requiring readmission (*p* = 0.031; Supplementary Table 4). Prevalence of death was 14% in benign patients and 56% in malignant patients (*p* < 0.001) during the study window. Median follow-up in the BC group was 27 months (7–65) and 13 months (3–33) in the malignant group (*p* < 0.05). When dividing malignant patients based on pathologic stage, BC patients (40%) still carried a greater rate of readmission regardless of whether pathologic stage was ≤ pT2 (13%; *p* < 0.001) or > pT2 (24%; *p* = 0.002).

**Table 3:** Postoperative complication classifications requiring readmission after cystectomy

Category	UTI/infection	Bleeding	Surgical	GU	GI	Wound	DVT/PE	Cardiac	Other	Neurologic	Pulmonary	Total
Benign	28 (37.3)	2 (2.7)	0 (0.0)	13 (17.3)	11 (14.7)	12 (16.0)	0 (0.0)	0 (0.0)	7 (9.3)	1 (1.3)	1 (1.3)	75
Malignant	29 (36.7)	1 (1.3)	2 (2.5)	16 (20.3)	13 (16.5)	5 (6.3)	4 (5.1)	4 (5.1)	1 (1.3)	0 (0.0)	4 (5.1)	79
Total	57	3	2	29	24	17	4	4	8	1	5	154

The following table categorizes postoperative complications after cystectomy between benign and malignant patients that led to readmission using the MSKCC Classification System. Total numbers with percentage of the total cohort in parentheses are displayed

ADI was significantly different between benign and malignant patients. Specifically, on multinomial logistic regression, ADI scores 1–4 (less socioeconomic deprivation) were significantly more likely in benign compared to malignant patients (*p* < 0.05; Supplementary Table 5). DD was similar between both groupings (*p* > 0.05).

## Discussion

This manuscript compares outcomes in a large data set between BC and RC for bladder cancer patients. Unsurprisingly, these patient populations had significant baseline demographic differences. Further, urinary diversion selection was different between these groupings, with ileal conduits more frequently utilized in the malignant population, fitting with previously published literature [21]. Additionally, postoperative complications immediately in-house, ED utilization, and readmission rates were greater in the BC population. SES was higher, based on ADI score, in BC patients. Taken together, these findings highlight that BC for non-oncologic disease and RC for bladder cancer patients has a significant variation in their baseline demographics, postoperative outcomes, and SES.

This study identified an in-house postoperative complication rate of 51% for BC patients, which was significantly greater than that of the malignant RC patients. Aissen et al. found that 60% of all patients who underwent BC experienced a postoperative complication in their study [9]. Osborn et al. reviewed 139 BC patients and noted a 57% complication rate postoperatively for Clavien grade ≥ II [22] The most common complications noted were blood transfusion, ileus, and pyelonephritis. Brown et al. compared BC with urinary diversion to BC without urinary diversion, and in the diverted group, GI, GU, and cardiac complications were most often seen [13]. Erpelding et al. noted a 30-day postoperative complication rate of 43% for BC in their study comparing BC patients to RC patients with bladder cancer [7] No difference was appreciated between the BC and malignant patients regarding complications in this study. Peñaranda et al. analyzed over 26,000 cystectomies, of which 1314 were for non-oncologic reasons [23] The authors found significantly greater rates of GU, infectious, and wound complications in the BC cohort. This is like our analysis, where we found that BC patients were more likely to have an in-house complication, with the most common reasons being GI, GU, or infectious. However, it is important to recognize that there was a greater proportion of continent diversions in the BC cohort, and evidence has shown that continent urinary diversions have greater rates of both GU and GI complications [24]. On multivariable regression, continent diversions were 5.1 times more likely in BC patients.

ED utilization and readmission rates were also analyzed. There is a paucity of data reporting ED utilization on BC. In the past, ED utilization for RC for bladder cancer has been estimated at around 7–34% [4, 5, 25]. In this study, ED use was significantly greater in BC patients, at 51% within 90 days of discharge from surgery relative to 34% in the malignant grouping. It is difficult to fully explain why BC patients presented to the ED at such a high rate, but given the increased incidence of postoperative complications, it is not necessarily surprising. Erpelding et al. found that readmission rates did not differ between BC and malignant RC patients in their comparative analysis [7]. The data presented in this study contradicts this, as BC patients were readmitted more frequently within 90 days of BC, at a rate of 40%. Interestingly, the most common cause of readmission was UTI/infectious. While the rate of UTI after BC is poorly understood, UTIs after RC are quite common, around 14–36% [26, 27]. In total, this data shows that BC utilizes ED resources and is more likely to be readmitted after discharge relative to patients undergoing RC for oncologic reasons.

As to the specific reasons why the BC population had greater complication rates after surgery, we feel it is multifactorial. Further, this is a paradox, as our analysis showed that BC patients often had more favorable characteristics than patients undergoing RC for malignancy. Many of these patients have neurologic disorders in accordance with neurogenic bladder, making postoperative care far more complex. Others have chronic pain syndromes like IC/BPS. We feel it is important to acknowledge the possibility that underlying systemic disease in the BC patient population plays a role in these results. Furthermore, as noted, the urinary diversion selection was not the same in the malignant cohort relative to the benign, with a far greater proportion of Indiana Pouches and neobladders performed in BC patients. In a study by Polm et al. examining long-term outcomes of Indiana Pouches in patients with non-oncologic conditions, 67% of patients required some form of surgical revision [28]. Monn et al. demonstrated Indiana Pouch patients have greater minor complication rates after cystectomy compared to ileal conduit patients [29]. While 90-day complication rates of BC with a neobladder are poorly assessed, overall neobladder complication rates have been estimated to near 58% within 90 days of RC for bladder cancer [30]. And, while there is likely not a unilateral cause for the higher rate of complications in BC patients, the significantly higher proportion of continent diversions likely played a role. We also divided the malignant cohort by pathological stage, to assess if this could have played a role in our results. However, regardless of stage, BC patients still had greater rates of complications, ED use, and readmission, going against notions that lower stage bladder cancer patients could be driving the results we identified. Regardless, these findings highlight the many potential reasons behind the differences we observed in complication rates between benign and malignant patients.

Prior publications have shown that the BC patient population and RC malignant population are not the same. These projects have often focused on comparing baseline demographics like age, gender, and comorbidities [9]. In this analysis, we too demonstrated that BC patients tended to be younger, female, and have fewer comorbidities with a lower mean CCI score. However, SES is well established to affect cystectomy outcomes, yet few have compared BC and oncologic RC patients [31]. Moreover, DD after RC has been associated with postoperative outcomes and survival [32]. Using ADI score as a surrogate for SES, better SES (lower ADI score) was observed in the BC grouping. This was confirmed on multivariable regression analysis. Given that BC patients had higher rates of in-house complications, ED use, and readmissions, this is somewhat unexpected, as it has long been assumed that lower SES patients strain the healthcare system more. No difference in DD was noted between BC and malignant RC patients, which is an interesting finding as BC patients did have higher complication rates after surgery prior to discharge.

Multiple limitations are worth consideration in this study. The retrospective nature opens the study design to selection bias. Additionally, all BC were performed via an open approach, whereas a significant portion of RC for bladder cancer were done robotically, and ideally, we would have a similar proportion of open and robotic surgeries done in each cohort. There are likely multiple confounding variables we were unable to fully capture in our data review that could impact our results. We attempted to adjust for this using a binary logistic regression but recognize this is not perfect. We also elected to delineate between in-house complications, postoperative ED visits, and complications causing readmission within 90 days of discharge, but these are arbitrary cutoffs and recognize others may disagree with this timeline. Further, there are likely other baseline patient characteristic differences between the BC cohort and malignant RC cohort which we did not capture in our review. This is particularly important as recent evidence has shown preoperative comorbidities, like prior thromboembolic events, affect the rate of complications after RC. [33]

## Conclusions

Research on cystectomy has often focused on those with malignancy, and benign surgery is often neglected. Despite having a lower CCI, benign cystectomy patients had greater rates of in-house complications, ED utilization postoperatively, and 90-day readmission rates. Further, the types of complications benign and malignant cystectomy patients had differed using the MSKCC classification system. We also demonstrated that the SES of patients presenting for benign cystectomy is greater than those undergoing cystectomy for malignant reasons. The causes of these differences call for additional investigation.

## Supplementary Information

Below is the link to the electronic supplementary material.

Supplementary file1 (DOCX 39 kb)

## Data Availability

The data used in this manuscript are not publicly available due to HIPAA compliance laws, but available in a limited de-identified format upon reasonable request to the corresponding author.
