# Supplementary material for: Comparing benign to malignant cystectomy: complications, emergency department utilization, readmissions, and socioeconomic status
Source: Int Urol Nephrol. 2025 Dec 25;58(7):2429–36. doi: 10.1007/s11255-025-04972-7 (PMC13309444; doi:10.1007/s11255-025-04972-7)
Supplement: Supplementary file 1 — Supplementary file1 (DOCX 39 kb) [file 11255_2025_4972_MOESM1_ESM.docx]

**Supplementary table 1. Binary logistic regression.** The following table is a binary logistic regression with either benign or malignant cystectomy as the outcome. Variables are shown with odds ratio, adjusted odds ratio, and confidence intervals. P-values are provided to show significance. Continuous variables are shown per unit increase (i.e. per year increase in age). Benign cystectomy is the reference category in this model.

| Variable | Odds ratio | Std. error | Significance | Adjusted odds ratio | Lower | Upper |
| --- | --- | --- | --- | --- | --- | --- |
| Female | 1.9 | 0.3 | <0.001 | 7.0 | 4.3 | 11.6 |
| Age (years) | -0.02 | 0.01 | 0.111 | 1.0 | 0.9 | 1.0 |
| CCI | 0.5 | 0.09 | <0.001 | 1.7 | 1.4 | 2.0 |
| ADI | - | - | 0.068 | - | - | - |
| 1 | -1.7 | 0.7 | 0.019 | 0.2 | 0.05 | 0.8 |
| 2 | -1.0 | 0.6 | 0.08 | 0.3 | 0.1 | 0.9 |
| 3 | -1.6 | 0.6 | 0.009 | 0.2 | 0.06 | 0.7 |
| 4 | -1.2 | 0.5 | 0.028 | 0.3 | 0.1 | 0.9 |
| ED visit within 90-days | -0.4 | 0.3 | 0.193 | 0.7 | 0.3 | 1.2 |
| Complication postoperatively | 1.1 | 0.3 | <0.001 | 2.8 | 1.7 | 4.7 |
| Readmission within 90-days | 1.4 | 0.4 | <0.001 | 4.1 | 2.0 | 8.3 |
| Continent urinary diversion | 1.6 | 0.5 | 0.001 | 5.1 | 1.9 | 13.9 |
| Constant | -3.4 | 0.9 | <0.001 | 0.03 | - | - |

**Supplementary table 2. Multinomial logistic regression for in-house complications.** The following table is a multinomial logistic regression with immediate in-house complications after cystectomy as the outcomes. Malignant patients are the referent group relative to benign cystectomy patients. B represents the odds ratio, std. error is the standard error, and Exp(B) is the adjusted odds ratio. For the model, “other complications” is the referent complication category. Complication categories that had no events in either the benign or malignant cohorts were excluded from the model (DVT/PE).

| Variable | B | Std. error | Significance | Exp(B) | Lower | Upper |
| --- | --- | --- | --- | --- | --- | --- |
| UTI/infection | 0.6 | 0.5 | 0.459 | 1.8 | 0.4 | 7.7 |
| Bleeding | -0.6 | 0.8 | 0.460 | 0.6 | 0.1 | 2.5 |
| Surgical | 1.5 | 1.0 | 0.148 | 4.5 | 0.6 | 34.6 |
| GU | 3.3 | 1.0 | <0.001 | 26.3 | 3.9 | 176.5 |
| GI | 0.8 | 0.7 | 0.232 | 2.3 | 0.6 | 8.5 |
| Wound | 0.8 | 0.8 | 0.305 | 2.3 | 0.5 | 10.6 |
| Cardiac | 1.7 | 0.8 | 0.027 | 5.8 | 1.2 | 27.1 |
| Neurologic | 1.0 | 0.9 | 0.283 | 2.6 | 0.5 | 15.3 |
| Pulmonary | 2.2 | 1.3 | 0.081 | 9.0 | 0.8 | 106.0 |
| Other | - | - | - | - | - | - |

**Supplementary table 3. Multinomial logistic regression for Clavien complications.** The following table is a multinomial logistic regression with Clavien grade complications after cystectomy as the outcomes. Malignant patients are the referent group relative to benign cystectomy patients. B represents the odds ratio, std. error is the standard error, and Exp(B) is the adjusted odds ratio. Associated p-values and confidence intervals are displayed. For the model, Clavien grade V is the referent complication category.

| Clavien | B | Std. error | Significance | Exp(B) | Lower | Upper |
| --- | --- | --- | --- | --- | --- | --- |
| I | -0.2 | 1.2 | 0.98 | 1.0 | 0.1 | 9.5 |
| II | -1.4 | 1.1 | 0.209 | 0.2 | 0.03 | 2.2 |
| IIIa | -1.9 | 1.2 | 0.106 | 0.1 | 0.01 | 1.5 |
| IIIb | -0.3 | 1.4 | 0.835 | 0.8 | 0.05 | 11.3 |
| IVa | -2.6 | 1.3 | 0.046 | 0.08 | 0.006 | 0.9 |
| IVb | -0.3 | 1.3 | 0.819 | 0.8 | 0.06 | 8.8 |
| V | - | - | - | - | - | - |

**Supplementary table 4. Multinomial logistic regression for readmission complications.** The following table is a multinomial logistic regression with immediate in-house complications after cystectomy as the outcomes. Malignant patients are the referent group relative to benign cystectomy patients. B represents the odds ratio, std. error is the standard error, and Exp(B) is the adjusted odds ratio. For the model, “other complications” is the referent complication category. Complication categories that had no events in either the benign or malignant cohorts were excluded from the model (surgical, DVT/PE, cardiac, and neurological).

| Variable | B | Std. error | Significance | Exp(B) | Lower | Upper |
| --- | --- | --- | --- | --- | --- | --- |
| UTI/infection | 2.0 | 1.1 | 0.072 | 7.3 | 0.8 | 62.8 |
| Bleeding | 1.9 | 1.8 | 0.272 | 7.0 | 0.217 | 226.0 |
| GU | 2.2 | 1.1 | 0.057 | 8.6 | 0.9 | 79.3 |
| GI | 2.1 | 1.1 | 0.065 | 8.3 | 0.9 | 78.0 |
| Wound | 1.0 | 1.2 | 0.406 | 2.7 | 0.3 | 27.8 |
| Pulmonary | 3.3 | 1.5 | 0.031 | 28.0 | 1.4 | 580.6 |
| Other | - | - | - | - | - | - |

**Supplementary table 5. Multinomial logistic regression for ADI.** The following table is a multinomial logistic regression with ADI as the outcome. Malignant patients are the referent group relative to benign cystectomy patients. B represents the odds ratio, std. error is the standard error, and Exp(B) is the adjusted odds ratio. For the model, an ADI score of 10 is the referent category.

| ADI | B | Std. error | Significance | Exp(B) | Lower | Upper |
| --- | --- | --- | --- | --- | --- | --- |
| 1 | -1.5 | 0.6 | 0.008 | 0.2 | 0.08 | 0.7 |
| 2 | -1.2 | 0.5 | 0.01 | 0.3 | 0.1 | 0.8 |
| 3 | -1.5 | 0.5 | 0.002 | 0.2 | 0.09 | 0.5 |
| 4 | -1 | 0.4 | 0.013 | 0.4 | 0.2 | 0.8 |
| 5 | -0.8 | 0.4 | 0.064 | 0.5 | 0.2 | 1.0 |
| 6 | -0.8 | 0.4 | 0.056 | 0.5 | 0.2 | 1.0 |
| 7 | -0.4 | 0.4 | 0.399 | 0.7 | 0.3 | 1.6 |
| 8 | -0.3 | 0.4 | 0.464 | 0.7 | 0.3 | 1.7 |
| 9 | -0.8 | 0.4 | 0.094 | 0.5 | 0.2 | 1.1 |
| 10 | - | - | - | - | - | - |
